# Supplementary material for: Genetic Parameters and Sex‐Specific Architecture of Observed and Latent Fertility Phenotypes in a Closed Breeding Nucleus of an Arctic Salmonid
Source: J Anim Breed Genet. 2026 Apr 22;143(5):667–78. doi: 10.1111/jbg.70051 (PMC13460882; doi:10.1111/jbg.70051)
Supplement: Supplementary file 1 [file JBG-143-667-s001.docx]

**Table S1.** Posterior metrics and convergence diagnostics for Fertilization Model 1.

| **Variable** | **Mean** | **SD** | $\hat{R}$ | **ESS_bulk** | **ESS_tail** |
| --- | --- | --- | --- | --- | --- |
| h^2^ for female fertility ($F_{f}$) | 0.158 | 0.045 | 1.00 | 2348 | 4703 |
| h^2^ for male fertility ($F_{f}$) | 0.047 | 0.043 | 1.00 | 1200 | 1388 |
| r_g_ | 0.050 | 0.266 | 1.00 | 9938 | 14172 |

$\hat{R}$is an indicator of convergence upon approaching 1. ESS denotes the effective sample size of the Markov chain iterations. ESS_bulk and ESS_tail are diagnostics for the sampling efficiency in the bulk and tail of the posterior. The former is defined as the effective sample size for rank normalized values using split chains, while the latter is defined as the minimum of the effective sample sizes for 5% and 95% quantiles.

**Table S2.** Posterior metrics and convergence diagnostics for Fertilization Model 2.

| **Variable** | **Mean** | **SD** | $\hat{R}$ | **ESS_bulk** | **ESS_tail** |
| --- | --- | --- | --- | --- | --- |
| h^2^ for female fertility ($F_{f}$) | 0.166 | 0.044 | 1.00 | 1830 | 3310 |
| h^2^ for male capacity ($C_{m}$) | 0.342 | 0.199 | 1.01 | 290 | 567 |
| r_g_ | -0.022 | 0.252 | 1.00 | 4697 | 8609 |

$\hat{R}$is an indicator of convergence upon approaching 1. ESS denotes the effective sample size of the Markov chain iterations. ESS_bulk and ESS_tail are diagnostics for the sampling efficiency in the bulk and tail of the posterior. The former is defined as the effective sample size for rank normalized values using split chains, while the latter is defined as the minimum of the effective sample sizes for 5% and 95% quantiles.
